# Genome Mining of *Streptomyces* sp. YIM 130001 Isolated From Lichen Affords New Thiopeptide Antibiotic

**DOI:** 10.3389/fmicb.2018.03139

**Published:** 2018-12-19

**Authors:** Olha Schneider, Nebojsa Simic, Finn Lillelund Aachmann, Christian Rückert, Kåre Andre Kristiansen, Jörn Kalinowski, Yi Jiang, Lisong Wang, Cheng-Lin Jiang, Rahmi Lale, Sergey B. Zotchev

**Affiliations:** ^1^Department of Biotechnology and Food Science, Norwegian University of Science and Technology, Trondheim, Norway; ^2^Department of Chemistry, Norwegian University of Science and Technology, Trondheim, Norway; ^3^Center for Biotechnology, Bielefeld University, Bielefeld, Germany; ^4^Yunnan Institute of Microbiology, Yunnan University, Kunming, China; ^5^Key Lab for Plant Diversity and Biogeography of East Asia, Kunming Institute of Botany, Chinese Academy of Sciences, Kunming, China; ^6^Department of Pharmacognosy, University of Vienna, Vienna, Austria

**Keywords:** new *Streptomyces* sp. from lichen, antibacterial activity, genome mining, new thiopeptide antibiotic, berninamycins

## Abstract

*Streptomyces* bacteria are recognized as an important source for antibiotics with broad applications in human medicine and animal health. Here, we report the isolation of a new lichen-associating *Streptomyces* sp. YIM 130001 from the tropical rainforest in Xishuangbanna (Yunnan, China), which displayed antibacterial activity against *Bacillus subtilis*. The draft genome sequence of this isolate strain revealed 18 putative biosynthetic gene clusters (BGCs) for secondary metabolites, which is an unusually low number compared to a typical streptomycete. Inactivation of a lantibiotic dehydrogenase-encoding gene from the BGC presumed to govern biosynthesis of a thiopeptide resulted in the loss of bioactivity. Using comparative HPLC analysis, two peaks in the chromatogram were identified in the extract from the wild-type strain, which were missing in the extract from the mutant. The compounds corresponding to the identified peaks were purified, and structure of one compound was elucidated using NMR. The compound, designated geninthiocin B, showed high similarity to several 35-membered macrocyclic thiopeptides geninthiocin, Val-geninthiocin and berninamycin A. Bioinformatics analysis of the geninthiocin B BGC revealed its close homology to that of berninamycins.

## Introduction

The successful use of any therapeutic agent is compromised by the potential development of tolerance or resistance to that compound from the time it is first deployed. This concerns all agents used in the treatment of bacterial, fungal, parasitic and viral infections, as well as cancer (Davies and Davies, 2010). Infectious diseases caused by bacteria are among the top causes of mortality in the world, and if appropriate actions are not implemented, it is estimated that by year 2050 up to 10 million people could die each year because of infections caused by antibiotic resistant pathogens (de Kraker et al., 2016). A successful curative treatment of such common, yet frequently deadly illnesses, as pneumonia and tuberculosis, could be soon history, if measures to tackle the antibiotic resistance problems are not implemented. Apart from the reduction of overuse and misuse of antimicrobial agents, identification of new antibiotics still plays an essential role in controlling bacterial infections.

The majority of antibiotics used in medicine, veterinary practice and agriculture originate from actinomycete bacteria, predominantly from those belonging to the genus *Streptomyces* (Barka et al., 2015). *Streptomyces* are Gram-positive bacteria with genomes of high GC content, widely distributed in terrestrial as well as in aquatic ecosystems, and have a complex life cycle, with a multicellular mycelial growth (Labeda et al., 2012). Their life cycle starts with the germination of a spore that grows out to form vegetative hyphae, and further differentiates from vegetative mycelium into the aerial mycelium. Morphological differentiation in *Streptomyces* is an intricately regulated process, which typically correlates with production of secondary metabolites (SMs), such as antibiotics (van Wezel and McDowall, 2011).

Thiopeptide antibiotics are a prominent class of antimicrobials with potent activity against Gram-positive bacteria, produced primarily by *Streptomyces* species (Bagley et al., 2005). The interest in this family of antibiotics was recently renewed, since many members of this class show activity against various drug-resistant pathogens, including methicillin-resistant *Staphylococcus aureus* (MRSA), penicillin-resistant *Streptococcus pneumoniae* (PRSP), and vancomycin-resistant enterococci (VRE) (Bagley et al., 2005). Recent success in generation of new thiopeptide analogs with significantly improved pharmacological properties may pave the way to the introduction of these types of compounds in drug development pipelines aimed at antibiotic resistant pathogens (Just-Baringo et al., 2014).

Thiopeptides belong to the group of ribosomally synthesized and post-translationally modified peptides (RiPPs) possessing unique thiazole rings and a six-membered tri-or tetra-substituted nitrogen heterocycle that can be present in one of the three oxidation states: a piperidine, dehydropiperidine, or pyridine. Biosynthesis of thiopeptides follows a relatively straightforward pathway: a precursor peptide consisting of an N-terminal leader and a C-terminal core peptide, encoded by a single gene, is synthesized and undergoes post-translational modifications. All thiopeptide biosynthetic gene clusters (BGC) contain, in addition to the gene encoding the precursor peptide, a set of at least five genes that encode enzymes required for heterocyclization, dehydration, and the formation of the central six-membered nitrogen heterocycle (Morris et al., 2009; Burkhart et al., 2017).

Advances in genetics, genomics and computer science have changed the way of natural product discovery. The complete genome sequencing of *Streptomyces coelicolor A3(2)* and *Streptomyces avermitilis* in early 2000s revealed a yet unexplored biosynthetic potential of the members belonging to this genus (Bentley et al., 2002; Ikeda et al., 2003) and led to the idea of genome-guided prediction and isolation of SMs, the approach known as genome mining (Du and van Wezel, 2018). Comparison of BGCs encoded in newly sequenced genomes using software such as antiSMASH (Blin et al., 2017) helps to identify conserved genes for enzymes involved in biosynthetic pathways for known classes of compounds, and thus to predict types of SMs they specify. In some cases, even the structure of the target SM can be predicted to a certain extent (Winter et al., 2011). To make the production, isolation and identification of SM faster and easier, the genome mining approach is combined with comparative metabolic profiling (Winter et al., 2011). Many different techniques contributed to the successful characterization of new compounds, such as heterologous expression (Yamada et al., 2015), regulatory genes overexpression or inactivation (Jiang et al., 2015; Chen et al., 2017), as well as knock-out of core scaffold-biosynthetic genes (Tian et al., 2016).

Here, we present the results of a genome mining linked to the metabolite profiling that led to identification of a new analog of the thiopeptide antibiotic geninthiocin, geninthiocin B, and its BGC from a *Streptomyces* sp. isolated from a lichen. By combining the classical bioassay-based screening with genome mining we could rapidly connect the thiopeptide BGC with the molecule it specifies.

## Materials and Methods

### Isolation and Identification of *Streptomyces* sp. YIM 13001

*Streptomyces* sp. YIM 130001 was isolated from a lichen *Lepidostroma yunnana* sp. nov. sample collected format the tropical rainy forest in Xishuangbanna (Yunnan, China) using YIM 212 medium (Raffinose 5.0 g, histidine 1.0 g, K_2_HPO_4_ 1 g, MgSO_4_⋅7H_2_O 0.5 g, Agar 15 g, water to 1 L, pH 7.2∼7.4) and incubation temperature of 28°C. Genomic DNA of YIM 130001 was isolated as described below, and used as a template to PCR-amplify 16S rRNA gene fragment with previously described primers and protocol (Bredholdt et al., 2007). The resulting DNA fragment was sequenced, and the sequence deposited in the GenBank under accession number MH532527. Phylogenetic analysis of the 16S rDNA sequence was performed using software MEGA 7.0 (Kumar et al., 2016).

### Genome Sequencing and Analyses

The genomic DNA isolation was done from a culture grown in 50 mL of 3% Tryptone Soya Broth medium (TSB, OXOID, United Kingdom) inoculated with 50 μL of freshly prepared spore suspension of YIM 130001 (20% glycerol, v/v) and incubated in 250 mL baffled flasks at 28°C, 250 rpm overnight. The genomic DNA was isolated using Wizard Genomic DNA Purification Kit (Promega, Madison, WI, United States) as described previously (Schaffert et al., 2016). For the genome sequencing, chromosomal DNA was used to generate a TruSeq PCR-free library that was sequenced on an Illumina MiSeq system in a 2 × 300 nt run. A total of 348.8 Mbp sequence data (43.5 × coverage) were assembled using NEWBLER version 2.8 (Roche), resulting in 59 scaffolds containing 80 contigs. Gene prediction and annotation were performed with PROKKA SOFTWARE (Seemann, 2014), the relevant genome features are listed in Supplementary Table S1. This Whole Genome Shotgun project has been deposited at the DDBJ/ENA/GenBank under the accession number QODG00000000. The version described in this paper is version QODG01000000.

### Generation of Recombinant Bacterial Strains, Plasmids, and General Growth Conditions

All routine DNA standard techniques, cloning methods, and plasmid transformation into *Escherichia coli* were performed as described in Sambrook et al. (1989). PCR fragment amplifications were done with Q5^®^ High-Fidelity DNA Polymerase (New England Biolabs, Ipswich, MA, United States) using oligonucleotides listed in Supplementary Table S2. Plasmids and bacterial strains used or constructed during this study are represented in Supplementary Table S3.

*Escherichia coli* strains were grown in Luria–Bertani (LB) broth or on LB agar, supplemented with chloramphenicol (25 μg mL^-1^), apramycin (100 μg mL^-1^), kanamycin (25 μg mL^-1^). XL1-blue strain was used for general cloning, ET12567 (pUZ8002) was used for intergeneric conjugative transfer of plasmids to *Streptomyces* as described before (Flett et al., 1997).

To inactivate the gene *genB*, a 1055 bps internal fragment from *genB* gene was amplified with primers genB_*Hind*III/genB_*EcoR*I primer pair from YIM 130001 genomic DNA and cloned into the 3.1 kb *EcoR*I/*Hind*III fragment of the vector pSOK201 (Supplementary Table S3). The generated plasmid pC1_KN was transferred into the YIM 130001 strain via conjugation and resulting *genB*-distruption mutant (YIM 130001/KN) was verified by PCR using genB_fwd/genB_rev primer pair (Supplementary Figure S1C). Wild-type strain YIM 130001 harboring empty vector pSOK804 was used as a control.

### Strain Fermentation and Extraction on a Small-Scale

The production of secondary metabolites by YIM 130001 was tested in the following liquid media: 5010 (g/L: sucrose 30.0, NaNO_3_ 2.0, KH_2_PO_4_ 1.0, MgSO_4_ × 7 H_2_O 0.5, KCl 0.5, FeSO_4_ × 7 H_2_O 0.01, pH 7.8): 5288 (g/L: glycerol 15.0, soy meal 10.0, NaCl 5.0, CaCO_3_ 1.0, CoCl_2_ × 6 H_2_O 0.001, pH 7.8); 5321 (g/L: peptone 10.0, glucose 20.0, CaCO_3_ 2.0, CoCl_2_ × 6 H_2_O 0.001, pH 7.8); 5333 (g/L: yeast extract 4.0, soluble starch 15.0, K_2_HPO_4_ 1.0, MgSO_4_ × 7 H_2_O 0.5, pH 7.8); and SM17 (Zettler et al., 2014). Based on the results from bioassay and HPLC, SM17 medium was chosen as most suitable for the production of antibacterial compound.

The pre-culture of strains YIM 130001, YIM 130001/pSOK806, and YIM 130001/KN were prepared from inoculation of 10 mL TSB medium, containing the 50 μg mL^-1^ apramycin for recombinant strains, with 50 μL of spore-suspension in 250 mL baffled flasks and cultivation for 24 h at 250 rpm, 30°C. Fifty milliliter of SM17 medium without apramycin was inoculated with 5% of pre-culture and was cultivated in 250 mL-baffled flasks for 5 days, 250 rpm at 30°C. The fermented broth was extracted with 50 mL of butanol (100%) by 250 rpm, at 30°C for 2 h. The separation of organic phase was done via centrifugation at 7000 rpm for 20 min Next, butanol was removed from the extract by rotor-evaporation at 45°C, yielding oily crude extract which was dissolved in 1 mL methanol (100%), filtrated through 0.22°μm sterile syringe filter (ThermoFisher Scientific) and used for bioassay and HPLC analysis.

### Bioassay of Antimicrobial Activity

The antibacterial activity of crude extract was tested by disk diffusion assay. Petri dishes containing 25 mL of nutrient agar media, depending on the bacterial species, were seeded with bacterial suspensions from glycerol stock (20% glycerol, v/v). The sterile Geade AA disk of Whatmann filter (6 mm in diameter) was impregnated with 15 μL of crude extract in methanol, dried for 15 min in the sterile bench and placed on the surface of the seeded agar plate. After 18 h of incubation the inhibition zone around the disk was measured. The antimicrobial properties of extracts were tested against *Bacillus subtilis*, *E. coli* XL1 Blue, and *Candida albicans* ATCC 10231. The strains *B. subtilis* and *E. coli* XL1 Blue, were grown in LB-medium for preparation of stock solutions (20% glycerol, v/v). The strain *C. albicans* ATCC 10231 was grown at 30°C for 18 h in M19 liquid medium (for glycerol stock solution, 20 % glycerol, v/v), or on M19 agar plates for bioassays (Hakvåg et al., 2008).

### Analytical Metabolomics Profiling of Extracts

Analytical RP HPLC was carried out with an Agilent 1290 HPLC system equipped with a diode-array UV detector (DAD) at 192–600 nm, and with the analytical column ZORBAX Eclipse XDB-C18 4.6 × 150 mm, 5 μm (Agilent). Elution was carried out with water containing formic acid (0.1%, v/v) as solvent A and acetonitrile as solvent B, with flow rate of 1 mL/min. The elution program used was: 0–47.5 min: linear gradient from 5 to 95% of solvent B, 47.5–50 min: isocratic 100% solvent B, 50–65 min: isocratic 5% solvent B.

### Production of Antimicrobial Compounds in a Bioreactor

Batch cultivation of YIM 130001 wild type strain was performed in 3 L bioreactor (BioFlo/CelliGen 115 Fermentor, New Brunswick Scientific) with a 1.5 L-working volume. The pre-culture was prepared by inoculation of 10 mL TSB medium with 50 μL of spore-suspension in 250 mL baffled flasks and cultivation for 24 h at 250 rpm, 30°C. Next, two 250 mL baffled flasks containing 50 mL TSB medium were inoculated with 5% of pre-culture and cultivated for 48 h at 250 rpm at 30°C to obtain the pre-culture for bioreactor inoculation. One point five liter of SM17 medium in bioreactor were inoculated with 75 mL of pre-culture and 1 mL of 10% silicone antifoam (stock solution: 30% in H_2_O, Sigma Aldrich) was added at the start of the fermentation. The dissolved oxygen level (DO) was kept above 40% using a stirring cascade from 300 to 1000 rpm, aeration rate from bioreactor was 0.75 vvm. The batch cultivation was carried out for 7 days at 30°C, without controlling pH value and foam formation in bioreactor. To prevent the foam formation, additional 1 mL of 10% silicone antifoam solution was added manually on fermentation days 3 and 5.

### Extraction and Purification of Antimicrobial Compounds

After 7 days of fermentation approximately 1.5 L of whole fermentation broth with the dry cell mass 58 g L^-1^ were extracted with 100% butanol (1:1 v/v), at 30°C and 250 rpm for 2 h. The separation of organic phase was done by centrifugation at 9000 rpm for 20 min. The oily crude extract (14.06 g) was obtained after removing the solvent in the rotavapor at 45°C. To remove the oily substances from the sample, to the crude extract was supplemented with 200 mL of 100% *n*-pentane, the mixture was well stirred and after 2 h of sedimentation at room temperature, the *n*-pentane phase was decanted and the pellet air-dried, yielding 8.06 g of air-dried material. Next, the pellet was dissolved in 50 mL of 100% methanol. The sample was purified using flash chromatography by gravity flow with silica gel 60 (0.063–0.200 mm, 70–230 mesh ASTM, Merck) as stationary phase. Mobile phases used were: fraction 1, ethyl acetate (100%), fraction 2, ethyl acetate/methanol (9:1, v/v), and fraction 3, ethyl acetate/methanol (8:2). All extractions and purifications steps were tested for bioactivity against *B. subtilis* and analytical RP HPLC as describe above. The fraction 2 (ca. 500 mL) showed the activity in the bioassay and the presence of expected peaks at 22 and 25 min in RP HPLC. The organic solvents from the fraction 2 were removed in rotavapor at 45°C, yielding 125 mg of material, which was dissolved in 1 mL of 100% methanol and subjected to preparative HPLC.

Preparative HPLC was carried out with an Agilent 1260 HPLC system equipped with a DAD and binary pump. Five hundred microliter of injected sample was separated on preparative column ZORBAX XDB-C18 21.2 × 150 mm 5 μm (Agilent). Elution was carried out with water containing formic acid (0.1%, v/v) as solvent A and acetonitrile as solvent B, with flow rate of 20 mL/min. The elution program used was: 0–47.5 min: linear gradient from 5 to 95% of solvent B, 47.5–50 min: isocratic 100% solvent B, 50–65 min: isocratic 5% solvent B. The fractions on retention time from 21.8 to 22.2 min (for compound 1) and 25.4–25.8 min (compound 2) were collected and freeze dried. Before freeze drying, the 15 μL of each sample was tested for bioactivity against *B. subtilis*.

### Flow Injection Analysis – qTOF Mass Spectrometry

Analyses were performed with an ACQUITY I-class UHPLC system, operating in flow injection analysis mode (FIA), coupled to a Synapt G2Si HDMS mass spectrometer (Waters, Milford, MA, United States) equipped with an ESI source operating in negative or positive mode.

Flow injection analysis analysis was performed by operating the UHPLC in bypass, in order to direct the flow passed the column compartment, and directly to the mass spectrometer. A mobile phase consisting of 100% methanol was used, and a linear flow gradient was programmed. The flow rate was constant at 0.150 mL min^-1^ for 0.10 min, then reduced to 0.030 min until 1.50 min, then increased to 0.200 mL min^-1^ until 1.60 min, and finally increased to 0.800 mL min^-1^ until 1.85 min. The system was equilibrated for additionally 0.25 min at 0.150 mL/min. The total run time was 2.0 min. The injection volume was set to 2 μL, and needle wash solvent consisted of 10% water in pure methanol. Sample compartment was cooled to 10°C before introducing the sample to the holder.

Mass spectrometric (MS) analyses were performed under constant ESI conditions. The capillary voltage, cone voltage and source offset voltage in negative and positive mode were set at -2.5 kV/3.0 kV, -30 V/30 V, and -40 V/40 V, respectively. The source temperature was maintained at 120°C, desolvation gas temperature 200°C, and desolvation gas flow rate was set at 800 L h^-1^. The cone gas flow rate was fixed at 50 L h^-1^ and the nebulizer gas flow maintained at 6.0 bar. The mass spectrometer was operated in scan mode. The resolution mode was set to continual high resolution. Scan time was 0.5 s, and inter-scan delay 0.015 s. Mass range acquired was 50–2000 Da, the same range as the valid calibration performed with Na-formate immediately before analysis using IntelliStart.

During the FIA analysis, a lockmass flow of 10 μL min^-1^ of leucine enkephalin (1 ng mL^-1^) was infused into the ion source to correct the mass axis on the fly. The lockmass flows from a separate capillary, and the capillary voltage of this capillary was set to 2.5 kV (negative or positive, depending on the operation mode).

UHPLC/FIA–qTOF data were acquired and processed using MassLynx software (v4.1). Elemental composition and isotope model features in MassLynx were used for identification.

### NMR-Based Structure Elucidation

The sample was prepared by dissolving 1.5 mg of compound in 160 μL DMSO-d6 (99.9% d) and transferred to a 3 mm NMR tube. All homo-and heteronuclear NMR spectra were recorded on a Bruker Ascend 800 MHz Avance III HD NMR spectrometer (Bruker BioSpin AG, Fälladen, Switzerland) equipped with 5 mm cryogenic CP-TCI probe. All NMR spectra were recorded at 25°C. Shifts were determined relative to TMS, using the residual DMSO signals for spectra calibration. For chemical shift assignment of compound, the following spectra were recorded: 1D 13C, 2D double quantum filtered correlation spectroscopy (DQF-COSY), 2D rotating-frame nuclear Overhauser effect correlation spectroscopy (ROESY) with 250 ms mixing time, 2D ^13^C heteronuclear single quantum coherence (HSQC) with multiplicity editing, 2D ^13^C HSQC-[^1^H,^1^H]TOCSY with 70 ms mixing time on protons, 2D heteronuclear multiple bond correlation (HMBC) with BIRD filter to suppress first order correlations and 2D ^15^N HSQC. The spectra were recorded, processed, and analyzed using TopSpin 3.5 software (Bruker BioSpin AG, Fälladen, Switzerland). Details on NMR-based structure elucidation and recorded NMR spectra are given in Supplementary Material.

## Results and Discussion

### Isolation and Preliminary Characterization of *Streptomyces* sp. YIM 130001

**FIGURE 1:**
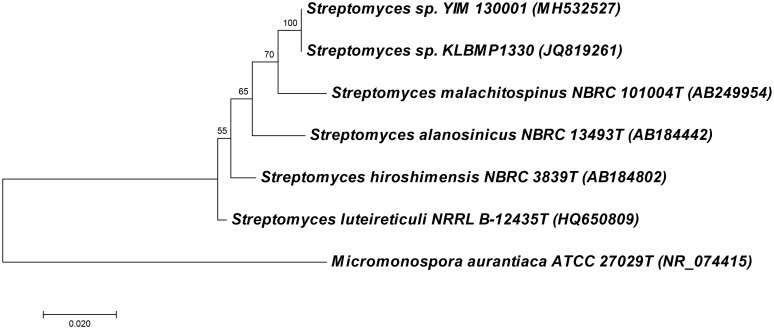
Molecular phylogenetic analysis of 16S rRNA gene fragments of *Streptomyces* sp. YIM 130001 and related species by Maximum Likelihood method. The evolutionary history was inferred by using the Maximum Likelihood method based on the Hasegawa-Kishino-Yano model. Evolutionary analyses were conducted in MEGA7 (19).

An actinomycete strain YIM 130001 was isolated from the lichen *Lepidostroma yunnana* sp. nov., collected at the rainy forest environment in Xishuangbann, province Yunnan in China. Based on the phylogenetic analysis analysis, this isolate was identified as *Streptomyces* sp. YIM 130001, with closest 16S rDNA sequence identity (98%) to that of the type strain *Streptomyces malachitospinus* NBRC 101004 (Figure 1). The best database match to the non-type strain was to the endophytic *Streptomyces* sp. KLBMP1330 isolated, among other actinomycetes, from the plant *Dendranthema indicum* (Linn.) Des Moul collected in East China (Xing et al., 2014).

The ability of YIM 130001 to produce antimicrobial compounds was tested by cultivating it in different types of media and testing culture extracts in bioassays (see “Materials and Methods” for details). It was shown that the *n*-butanol extract from the culture grown in SM17 medium exhibits bioactivity against *B. subtilis*, while no effect was shown on the growth of *E. coli* and *C. albicans*.

### Analysis of the *Streptomyces* sp. YIM 130001 Genome

Due to the fact that *Streptomyces* sp. YIM 130001 appeared to be phylogenetically diverse from the closest type strain and was bioactive, we decided to obtain its genome sequence in order to reveal secondary metabolite biosynthesis potential of this strain. The high-quality draft genome sequence of *Streptomyces* sp. YIM 130001 revealed a single linear chromosome of 8,025,327 bp, with no plasmids, and a G+C content of 70.75% (see Supplementary Table S1, Supplemental Material, for additional genome features). Analysis of the YIM 130001 genome sequence using antiSMASH 4.1.0 software (Blin et al., 2017) revealed 17 putative BGCs for the biosynthesis of secondary metabolites (Table 1), which is an unusually low number compared to a typical streptomycete with a 8-Mb genome. Whole genome sequencing of multiple *Streptomyces* species typically reveals between 25 and 50 BGCs per genome (Katz and Baltz, 2016).

**Table 1:** Secondary metabolite BGCs in *Streptomyces* sp. YIM 130001 predicted with antiSMASH 4.1.0 followed by manual curation with Protein BLAST and MIBiG algorithms.

Cluster no.	Cluster type	Best database cluster hit	Putative product
1	Thiopeptide	*Streptomyces atroolivaceus* NRRL ISP-5137	Geninthiocin B
2	T3pks	no plausible hits	Polyketide
3	Terpene	no plausible hits	Terpenoid
4	Terpene	*Streptomyces mobaraensis* NBRC 13819	Geosmin
5	Ectoine	*Streptomyces anulatus*	Ectoine
6	Bacteriocin	*Streptomyces turgidiscabies* Car8	Bacteriocin-like
7	Bacteriocin	no plausible hits	Bacteriocin-like
8	Bacteriocin	no plausible hits	Likely false
9	T1pks-Otherks	*Streptomyces albus* BK3-25	Polyketide, glycosylated
10	Nrps	Many *Streptomyces* spp.	Griseobactin/ Bacillibactin-likesiderophore
11	Nrps-Linaridin	no plausible hits	NRPS-PKS hybrid product
12	T3pks-Transatpks-Terpene-Otherks-Nrps	*Streptomyces* sp. CB01635	Streptogramins
13	Terpene	*Streptomyces* sp. Root55	Hopene
14a 14b	Terpene Nrps	*Streptomyces phaeoluteigriseus Streptomyces* sp. Wb2n-11	Isorenieratene NRS peptide
15	Siderophore	Many *Streptomyces* spp.	Acinetoferrins
16	Lassopeptide	Many *Streptomyces* spp.	Putative Class II lasso peptide
17	T1pks	*Streptomyces sp.* CB02414	Enediyne


Seven of the clusters identified in YIM 130001 genome contain genes encoding modular enzymes, such as polyketide synthases (PKS) and non-ribosomal peptide synthetases (NRPs). Cluster 17, a PKS type I BGC, was predicted to govern biosynthesis of a putative enediyene (Table 1). Up to now, only 12 natural enediyenes were characterized and two of them are approved as anticancer drugs and at least other four are in various stages of drug development, making this compound class attractive for drug discovery (Gredičak and Jerić, 2007). Cluster 10 was predicted to contain NRPS-encoding genes, which putative product is a griseobactin/bacillibactin-like siderophore. Four clusters were annotated as hybrid BGCs: the putative products from clusters 9 and 11 could not be predicted, while the BGC 12 has a homology to the cluster specifying streptogramins biosynthesis. Streptogramin-like antibiotics occur as two structurally different compounds that act synergistically to inhibit ribosomal peptidyl transfer during bacterial protein biosynthesis, while separately they exhibit only moderate antibacterial activity (Di Giambattista et al., 1989). antiSMASH initially predicted cluster 14 as being of terpene-NRPS hybrid type. However, closer inspection using BLAST and genetic context suggested that this locus is likely to comprise two separate BGCs, 14a for a carotenoid, and for 14b non-ribosomally synthesized peptide (Table 1).

In addition to the PKS- and NRPS- containing gene clusters, three BGCs for terpenoid biosynthesis were identified, one of them presumably responsible for hopanoids synthesis (Table 1). Hopanoids have a condensing effect on biological membranes due to their rigid ring structures, which help to stabilize the membranes once integrated. In *S. coelicolor* hopanoids are synthesized during formation of aerial hyphae to alleviate stress in aerial mycelium by diminishing water permeability across the membrane (Poralla et al., 2000). Cluster 16 was predicted to encode a putative lassopeptide, whereby manual inspection with Protein BLAST and MIBiG algorithms showed similarity to BGCs in many *Streptomyces* spp. The genes from cluster 15 showed high similarity (59–93%) to the genes from many other *Streptomyces* spp. that encode putative siderophore actinoferrin involved in iron acquisition (Sandy and Butler, 2009). A likely product from cluster 5 was predicted as ectoine, its BGC has high similarity with its counterpart in *Streptomyces anulatus*. Ectoines are most commonly found osmolytes in *Streptomyces* and help microorganisms to cope with osmotic stress (Sadeghi et al., 2014). Three BGCs were predicted to encode bacteriocins-like compounds. These clusters showed high similarity to other conserved clusters, the metabolic products of which are not known. Cluster 8, due to sequence gaps within the genome, was likely falsely annotated as a BGC (Table 1). The cluster 1 was predicted by antiSMASH software as a thiopeptide BGC, and after the manual Protein BLAST search, its high homology to the berninamycin gene cluster (Malcolmson et al., 2013) was noticed.

### Analysis of Thiopeptide BGC and Identification of the Cognate Bioactivity

**FIGURE 2:**
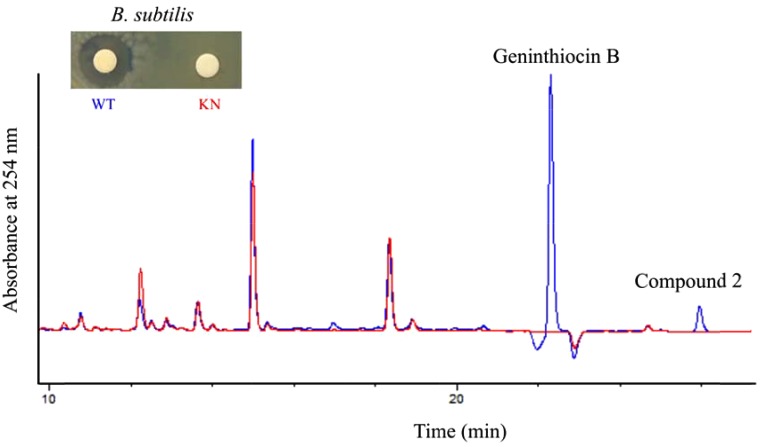
Comparative RP-HPLC chromatogram at 254 nm of the crude extracts from *Streptomyces* sp. YIM 130001 wild type (WT, in blue) and the strain YIM 130001/KN with deactivated *genB* gene (KN, in red), with corresponding bioassay against *B. subtilis*.

Among the clusters identified in the *Streptomyces* sp. YIM 130001 genome, cluster 1 was predicted by antiSMASH to specify biosynthesis of a thiopeptide, and manual inspection of encoded gene products using BLAST predicted that it could yield a berninamycin-like analog (Table 1). Due to the fact that the members of thiopeptide family are known for their antimicrobial activities against Gram-positive bacteria (Just-Baringo et al., 2014), we assumed that the product of cluster 1 might be responsible for the activity against *B. subtilis* in crude extract. In order to investigate this, *genB* gene encoding a putative lantibiotic dehydratase (Table 2) was inactivated via insertion of the pC1_KN vector into its coding region, yielding recombinant strain YIM 130001/KN. The correct insertion of the knock-out vector was subsequently confirmed by PCR (Supplementary Figure S1, Supplemental Material). The crude butanol extract from recombinant strain YIM 130001/KN was tested for antibacterial activity against *B. subtilis* and its metabolite profile was analyzed with analytical RP-HPLC in comparison with a butanol extract from the wild-type strain (Figure 2). YIM 130001/KN-extract, in contrast to that from the wild-type strain, failed to exhibit antibacterial activity against *B. subtilis*. Furthermore, using comparative analytical RP-HPLC it was shown that in the extract of YIM 130001/KN strain two chromatographic peaks at ca 22 and 25 min disappeared (Figure 2), indicating that those two peaks might correlate with both the antibacterial activity and the cluster 1 product.

**Table 2:** Deduced functions of ORFs in and around the geninthiocin B biosynthetic gene cluster.

Gene no.	Size of protein^a^	Protein homolog and its source	Accessions number	Identity (%)	Proposed function
*orf 1*	1304	β’-subunit DNA-directed RNA polymerase; *Streptomyces scopuliridis*	WP_030351424.1	97	Transcription of DNA into RNA
*orf 2*	149	*N*-acetyltransferase; *Streptomyces eurocidicus*	WP_102917203.1	71	Transfer of acetyl groups from acetyl-CoA
*orf 3*	123	S12; Many *Streptomyces* sp.	WP_007265893.1	99	30S ribosomal protein
*orf 4*	156	S7; *Streptomyces tsukubensis*	WP_077968976.1	99	30S ribosomal protein
*orf 5*	706	EF-G; *Streptomyces rapamycinicus* NRRL 5491	AGP55834.1	93	Elongation factor
*orf 6*	397	EF-Tu; *Streptomyces tsukubensis*	WP_077968978.1	98	Elongation factor
*genE1*	234	hypothetical protein SAMN05444921_112167; *Streptomyces wuyuanensis*	SDM72766.1	76	Thiazoline dehydrogenase
*genE2*	543	TpaE; *Streptomyces wuyuanensis*	WP_093656415.1	71	Thiazoline dehydrogenase
*genG1*	212	hypothetical protein; *Streptomyces atroolivaceus*	WP_051709133.1	71	Bacteriocin biosynthesis cyclodehydratase domain
*genG2*	448	hypothetical protein; *Streptomyces atroolivaceus*	WP_051709136.1	82	Thiazole-forming peptide maturase
*genD*	357	BerD; *Streptomyces bernensis*	AGN11669.1	61	Putative pyridine-forming enzyme
*genA*	46	thiocillin/thiostrepton family thiazolyl peptide; *Streptomyces wuyuanensis*	WP_094030676.1	86	Geninthiocin B structural gene
*genB*	885	hypothetical protein; *Streptomyces atroolivaceus*	WP_051709141.1	78	Lanthibiotic dehydratase N-terminus
*genC*	320	hypothetical protein; *Streptomyces atroolivaceus*	WP_078597759.1	76	Lanthibiotic dehydratase C-teminus
*genH*	400	cytochrome P450; *Streptomyces atroolivaceus*	WP_033297595.1	83	Cytochrome P450
*genI*	114	hypothetical protein; *Streptomyces wuyuanensis*	WP_093656403.1	86	C-terminal amide-forming enzyme
*orf 7*	102	S10; Many *Streptomyces* sp.	WP_014054162.1	100	30S ribosomal protein
*orf 8*	214	L3; *Streptomyces varsoviensis*	WP_030890763.1	98	50S ribosomal protein
*orf 9*	215	L4; *Streptomyces indicus*	WP_093613746.1	94	50S ribosomal protein
*orf 10*	106	L23; *Streptomyces indicus*	WP_093613748.1	96	50S ribosomal protein


*^*a*^Numbers refer to amino acid residues.*

### Purification and Structure Elucidation of Bioactive Compounds Produced by *Streptomyces* sp. YIM 130001

An up-scaled fermentation of YIM 130001 in medium SM17, followed by multiple steps of silica gel and preparative C-18 chromatography (see “Materials and Methods” for details), afforded two compounds, compound 1 (1.5 mg) and compound 2 (0.1 mg), both with antibacterial activities against *B. subtilis* (data not shown). During this study, the optimal conditions to elicit production of compound 2 were not developed, and a sufficient amount could not be purified to perform a proper MS/MS analyses or structure elucidation via NMR. The molecular masses of compounds 1 and 2 were determined on the basis of qTOF mass spectrometry, that afforded the masse-to-charge ratios of 1062.2965 *m/z* and 1046.3030 *m/z* (1061.2965 *m/z* and 1045.3030 *m/z* in negative mode, Supplementary Figure S2), respectively.

**FIGURE 3:**
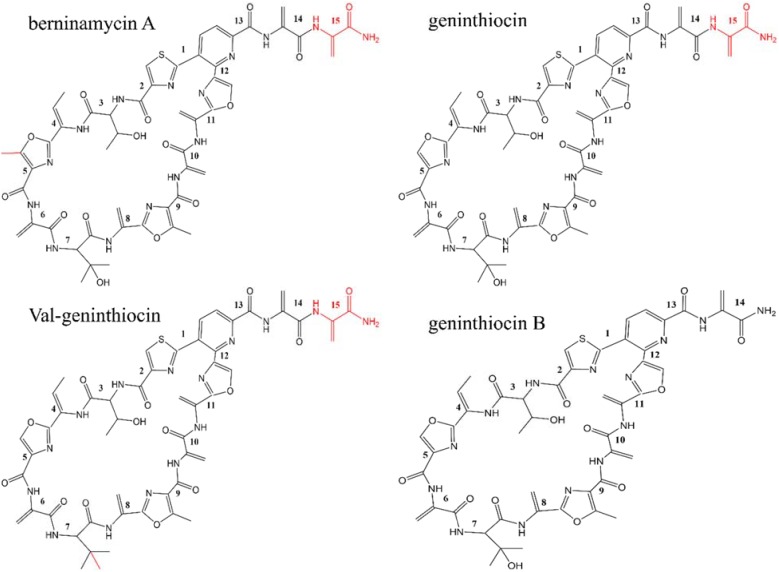
Chemical structures of the newly isolated geninthiocin B and its analogs berninamycin A, geninthiocin, and val-geninthiocin.

The NMR structure elucidation of compound 1 showed clearly that the molecule contained a free primary amido-group (N-56, Supplementary Figure S3) and eight peptide bonds (Supplementary Table S4). In addition, five terminal double bonds indicated five didehydroalanine residues. Threonine residue, didehydrobutyrine and hydroxy valine were also easily identifiable. Identification of cyclic structures of thiazol, oxazol, and pyridine rings were somewhat more challenging because of relatively large number of non-protonated carbons/heteroatoms and distant protons. By combining data from DQF-COSY, HMBC, HSQC, ROESY, and ^15^N-HSQC, the connectivity between the previously identified fragments was established. Protons from two OH groups could not be detected and assigned, probably because of their strong involvement in H-bonding. The shift assignments of ^1^H, ^13^C, and ^15^N (protonated) are shown in Supplementary Table S4. Based on NMR results, the molecular formula of compound 1 was determined as C_47_H_46_N_14_O_14_S, and the elucidated chemical structure indicated it closely resembles thiopeptide geninthiocin (Yun et al., 1994), albeit differing from the latter at the C-terminal part (Figure 3). Consequently, compound 1 was designated as geninthiocin B. The scaffolds from geninthiocin-like thiopeptides (geninthiocin and val-geninthiocin) are very similar to berninamycins (e.g., berninamycin A), which differ by one additional methyl-group on the oxazol ring (Figure 3). At the same time, geninthiocin B differs from two up to now characterized geninthiocins (Yun et al., 1994; Sajid et al., 2008) in lacking one Dha-residue on its C-terminus tail region. The macrocycle of newly discovered geninthiocin B is 35-membered, and because of its tri-substituted pyridine ring, it belongs to the series *d* thiopeptides (Just-Baringo et al., 2014).

### Analysis of the Geninthiocin B Biosynthesis Gene Cluster

**FIGURE 4:**
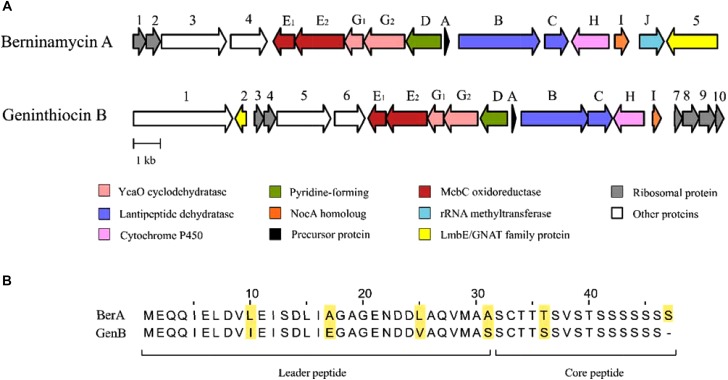
The biosynthetic gene clusters for berninamycin A and the newly isolated geninthiocin B **(A)**, with their precursor peptide sequences **(B)**, encoded by the structural genes. In the precursor peptide sequence, the core peptide appears in the mature thiopeptide, while the leader peptide sequence is removed during posttranslational modification.

The geninthiocin B biosynthesis gene cluster spans ca. 23.4 kb and contains 20 open reading frames (Table 2, Figure 4). BLAST analysis of this thiopeptide BGC showed similarity to *Streptomyces atroolivaceus* and *Streptomyces wuyuanensis* uncharacterized clusters. Its genes, especially their organization, were predicted to have a similarity to the BGC for berninamycin biosynthesis (Figure 4, Malcolmson et al., 2013). However, the comparison of the GenA core peptide sequence showed a closer similarity to geninthiocin isolated from *Streptomyces* sp. DD84 (Yun et al., 1994) and val-geninthiocin, originated from *Streptomyces* sp. RSF18 (Sajid et al., 2008). Up to now, only a few thiopeptides with 35-membered macrocycles have been described: geninthiocin, berninamycin, sulfomycin (Abe et al., 1988), thioplabin (Ohyama et al., 2002), and TP-1161 (Engelhardt et al., 2010a), and respective BGCs were reported for TP-1161 and berninamycin (Engelhardt et al., 2010b; Malcolmson et al., 2013). The newly isolated geninthiocin B is another member of this thiopeptide group and its identified BGC helps to understand its biosynthesis based on the homology to that of berninamycin (Figure 4).

Burkhart et al (2017) suggested that due to the structural similarity between geninthiocin and berninamycin, the biosynthesis of those two compounds is likely to be very similar. *In silico* prediction of enzymatic functions of individual gene products from genenthiocin B BGC allowed us to propose the biosynthesis of geninthiocin B (Supplementary Figure S4). The geninthiocin B structural gene (*genA*) encodes a 31-aa leader peptide linked to a C-terminal 15-aa core (Figure 4). Ten amino acids of the core peptide are represented by serine, whereby Ser_15_ is absent in the mature scaffold (Figure 3). The cleavage of Ser_15_ is needed to afford the C-terminus amide, and is presumably catalyzed by GenI, which is homologous to the NocA and NosA enzymes from nocathiacin and noshipeptide biosynthesis. It has been shown that NosA acts on an intermediate bearing a bis-dehydroalanine tail in noshipeptide and catalyzes an enamide dealkylation to remove the acrylate unit originating from the extended serine residue (Yu et al., 2010; Wang et al., 2015).

As in the berninamycin BGC, the unique feature of geninthiocin B cluster is that its genes for cyclodehydratase and dehydrogenase (most likely responsible for the formation of thiazole/oxazole rings), are split in a non-traditional way (Figure 4). The geninthiocin B biosynthesis YcaO “*G*” protein is split into GenG1/GenG2 and the protein “*E*” is split into GenE1/GenE2. The protein GenE2 probably has the partner function of the so-called E1-like proteins, which interact with a side chain of the leader peptide and promote GenG1 and GenG2 to perform the *d* cyclodehydration. In earlier studies it was observed that E1-like partner protein is required for substrate guiding and recognition by YcaO protein (Burkhart et al., 2015), as well as playing a crucial role in allosteric activation of the latter (Dunbar et al., 2012, 2014). This partner protein can either be represented by a discrete polypeptide or fused to the YcaO (Burkhart et al., 2017). Similarly to the berninamycin BGC, unusual is an appearance of the canonical E1-like protein fused to a dehydrogenase (GenE2), as well as the presence of another dehydrogenase component (GenE1) encoded by the gene near *genE2*. Due to this atypical genetic organization, it is unclear how the active azole-forming proteins function with each other. Burkhart et al. (2017) suggested that these proteins might catalyze the formation of a large 35-member macrocycle, since the spacing of azole/azoline heterocycles in the macrocycle region of berninamycin is nearly identical to that of the thiocillin-like thiopeptides, which have a 26-membered macrocycle after maturation.

Proteins GenB and GenC (Table 2) showed high similarity to lanthipeptide-like dehydratases and most likely catalyze the formation of the dehydroalanine (Dha) and dehydrobutyrine (Dhb) functional groups. Reconstitution studies using purified enzymes have established both the nature and the timing of the modifications installed during the biosynthesis of the thiopeptide antibiotic thiomuracin in *Thermobispora bispora* (Zhang et al., 2016). It was shown that after formation of the Dha residues, the central six-membered nitrogenous heterocycle is assembled via a formal [4+2] cycloaddition between the two Dha groups (Zhang et al., 2016). We suggest that in geninthiocin B biosynthesis the serine Ser_1_ and Ser_13_ are the key amino acids in the final macrocycle formation (Figure 4). GenD protein showing high degree of similarity to BerD from berninamycin pathway, which was predicted to catalyze the formation of characteristic for thiopeptides series *d* tri-substituted pyridine ring, is most probably also involved in the macrocycle formation (Malcolmson et al., 2013).

The gene *genH* (Figure 4, Table 2) encodes a homolog of BerH from berninamycin BGC, a putative P450 hydroxylase family protein that is most probably involved in valine hydroxylation (Malcolmson et al., 2013). The characterization of two P450 cytochrome-like proteins of nosiheptide (NosB and NosC) in *Streptomyces actuosus* ATCC 25421 indicated that those are responsible for hydroxylation occurring most likely at the tailoring stage, after the main scaffold is formed (Liu et al., 2013). During this work we could detect two compounds: geninthiocin B and an uncharacterized compound 2, both products from the geninthiocin B gene cluster. Based on the high-resolution LC-MS analysis, the compound 2 differs from geninthiocin B by one oxygen atom (Supplementary Figure S2). We suggest that compound 2 has similar structure to geninthiocin B, but lacks a hydroxyl group on Val_7_-residue. The appearance of a thiopeptide intermediate is not surprising and was described before for berninamycin (berninamycins A and B) (Lau and Rinehart, 1994). Moreover, a desoxygeninthiocin (val-geninthiocin) was isolated from *Streptomyces* sp. RSF18, which has a similar structure as geninthiocin, but lacks a hydroxyl-group on Val_7_-residue. If our assumption for structural features of compound 2 is correct, it can be produced as a major metabolite from the geninthiocin B cluster upon inactivation of the *genH* gene. The overall yield of geninthiocin B-related compounds must be improved to allow for more detailed studies on these molecules. The latter can be achieved either via heterologous expression of the cluster in an engineered *Streptomyces* host (Myronovskyi et al., 2018), or re-factoring of the cluster in YIM 130001 via promoter replacement (Horbal et al., 2018).

Geninthiocin B gene cluster is flanked at both ends by the genes encoding ribosomal and translation-associated proteins (Figure 4, Table 2), which are presumably not involved in geninthiocin B and compound 2 biosynthesises, but may play a key role in self-resistance of *Streptomyces* sp. YIM 130001. Berninamycin, which like genintiocin B also possesses a 35-membered macrocycle, is known to target 50S ribosomal subunit by binding to the 23S/L11 complex, thereby inhibiting protein synthesis (Reusser, 1969; Thompson et al., 1982). The mechanisms of self-resistance to 35-membered macrocyclic thiopeptides are still not completely understood (Just-Baringo et al., 2014), but for the berninamycin-producing *Streptomyces bernensis* it was shown that its respective BGC contains a gene encoding ribosomal RNA methylase. These proteins catalyze specific methylation of ribose on the 23S ribosomal RNA, preventing binding of thiopeptides to the modified ribosomes (Thompson et al., 1982). No genes encoding putative ribosomal RNA methylase could be identified in the geninthiocin B gene cluster or its flanking regions, suggesting that self-resistance in YIM 130001 may be achieved via different mechanism. This may also imply that the molecular target of geninthiocin B is different from that of berninamycin, despite very close similarity between their chemical structures and the same size of the macrocycle.

It has been speculated that some thiopeptides, including thiostreptone commonly used in veterinary medicine and research, may also serve at sub-inhibitory concentrations as signal molecules for physiological development or quorum-sensing in bacteria (Holmes et al., 1993). Keeping in mind the fact that thiopeptides at higher concentration have strong antibacterial activity (Bagley et al., 2005), a likely other ecological function of geninthiocin B could be defense against predatory bacteria and/or means of competition for nutritional sources.

## Conclusion

Streptomycetes are characterized by a complex metabolism and their ability to produce diverse types of antibiotics. In this work we describe the identification of new thiopeptide antibiotic, geninthiocin B, from *Streptomyces* sp. YIM 130001 with bioactivity against *B. subtilis*. Combined analysis of the genome sequencing data and metabolite profiling led to identification of geninthiocin B gene cluster, confirming the power of genome mining approach in the natural product discovery process.

## Author Contributions

LW, YJ, and C-LJ isolated and characterized *Streptomyces* sp. YIM 130001. SZ designed the experiments and supervised the study. OS, NS, FA, RL, and KK performed the wetlab experiments. CR and JK sequenced, annotated and analyzed the genome.

## Conflict of Interest Statement

The authors declare that the research was conducted in the absence of any commercial or financial relationships that could be construed as a potential conflict of interest.
