# Supplementary material for: Genome Mining of Streptomyces sp. YIM 130001 Isolated From Lichen Affords New Thiopeptide Antibiotic
Source: Front Microbiol. 2018 Dec 19;9:3139. doi: 10.3389/fmicb.2018.03139 (PMC6306032; doi:10.3389/fmicb.2018.03139)
Supplement: Supplementary file 1 [file Data_Sheet_1.docx]

**Supplemental Material**

**Genome Mining of *Streptomyces* sp. YIM 130001 Isolated from Lichen Affords new Thiopeptide Antibiotic**

Olha Schneider^1^, Nebojsa Simic^2^, Finn Lillelund Aachmann^1^, Christian Rückert^3^, Kåre Andre Kristiansen^1^, Jörn Kalinowski^3^, Yi Jiang^4^, Lisong Wang^5^, Cheng-lin Jiang^4^, Rahmi Lale^1^, Sergey B. Zotchev*^6^

**Table S1.** *Streptomyces* sp. YIM 130001 genome features

| **Genome Feature** | **Value** |
| --- | --- |
| Genome size (bp) | 8,025,327 |
| No. of contigs | 80 |
| No. of scaffolds | 59 |
| No. of genes | 7,083 |
| No. of CDSs | 7,003 |
| - with function prediction | 5,121 |
| - with signal peptide | 652 |
| No. of t(m)RNA genes | 75 |
| No. of other ncRNA features | 27 |
| G+C mol % | 70.75 |

**Table S2**. Oligonucleotides used in this study (Italic: endonuclease restriction enzyme site)

| Oligonucleotide | Oligonucleotide sequence |
| --- | --- |
| *genB*_HindIII | CGATAAG*AAGCTT*TGCCCTCGGGAGGCCACCAG |
| *genB*_EcoRI | GAAAGC*GAATTC*TGCTCACGCTGGGCGGTCTG |
| *genB*_fwd | ACTGCTCGTGAACTGGGTACTC |
| *genB*_rev | GATCCGCTCGAACCTCCAGATG |

**Table S3**. Plasmids and strains used in this work

| **Plasmid** | **Description** | **Source** |
| --- | --- | --- |
| pSOK806 | ColE1replicon origin, Am^R^, RP4 *ori*T*,attP*_,_*int*, *ermE**p | (1) |
| pSOK201 | pSG5 minimal replicon, Am^R^, RP4 oriT, ColEI replication origin | (2) |
| pC1_KN | Suicide plasmid for disruption of the genB gene, Am^R^ | This work |
| **Bacterial strain** | **Description** |  |
| *E. coli* XL1 Blue | general cloning host | New England Biolabs |
| *E. coli* ET12567/pUZ8002 | strain for intergenic conjugation; Km^R^, Cm^R^ | (3) |
| YIM 130001 | Wild-type | This work |
| YIM 130001/pSOK806 | Wild-type, harbouring empty pSOK806 vector | This work |
| YIM 130001/KN | Wild-type with integrated pC1_KN vector for *genB* gene disruption | This work |

**Table S4.** Assigned ^1^H, ^13^C and protonated ^15^N shifts of the thiopeptide

| **No.** | **δ ^13^C** | **δ ^1^H** |  | **δ ^15^N^[[1]](#footnote-1)^*** | **δ ^1^H** |  |
| --- | --- | --- | --- | --- | --- | --- |
| 1 | 146.8 | - |  |  |  |  |
| 2 | 130.3 | - |  |  |  |  |
| 3 | 140.9 | 8.53 (d, *J*=8.1) |  |  |  |  |
| 4 | 121.5 | 8.25 (d, *J*=8.1) |  |  |  |  |
| 5 | 149.5 | - |  |  |  |  |
| 6 | 163.1 | - |  |  |  |  |
| 7 | 126.9 | 8.50 (s) |  |  |  |  |
| 8 | 149.4 | - |  |  |  |  |
| 9 | 159.9 | - |  |  |  |  |
| 10 |  |  |  | 107.9 | 8.03 (d, *J*=8.8) |  |
| 11 | 57.8 | 4.61 (dd, *J*=3.3, 8.8) |  |  |  |  |
| 12 | 67.3 | 4.29 (m) |  |  |  |  |
| 13 | 20.5 | 1.15 (d, *J*=6.3) |  |  |  |  |
| 14 | 168.8 | - |  |  |  |  |
| 15 |  |  |  | 117.0 | 9.66 (bs) |  |
| 16 | 123.1 | - |  |  |  |  |
| 17 | 129.6 | 6.56 (m) |  |  |  |  |
| 18 | 13.8 | 1.75 (d, *J*=7.1) |  |  |  |  |
| 19 | 159.4 | - |  |  |  |  |
| 20 | 142.8 | 8.71 (s) |  |  |  |  |
| 21 | 136.1 | - |  |  |  |  |
| 22 | 158.4 | - |  |  |  |  |
| 23 |  |  |  | 123.0 | 9.39 (bs) |  |
| 24 | 133.4 | - |  |  |  |  |
| 25 | 103.8 | 5.89 (bs)  6.46 (bs) |  |  |  |  |
| 26 | 163.7 | - |  |  |  |  |
| 27 |  |  |  | 108.9 | 8.30 (d, *J*=8.4) |  |
| 28 | 61.8 | 4.64 (d, *J*=8.4) |  |  |  |  |
| 29 | 74.0 | - |  |  |  |  |
| 30 | 26.2 | 1.21 (s) |  |  |  |  |
| 31 | 27.3 | 1.22 (s) |  |  |  |  |
| 32 | 169.4 | - |  |  |  |  |
| 33 |  |  |  | 128.1 | 9.70 (bs) |  |
| 34 | 128.6 | - |  |  |  |  |
| 35 | 105.7 | 5.65 (bs)  6.11 (bs) |  |  |  |  |
| 36 | 155.2 | - |  |  |  |  |
| 37 | 129.2 | - |  |  |  |  |
| 38 | 154.5 | - |  |  |  |  |
| 39 | 11.5 | 2.62 (s) |  |  |  |  |
| 40 | 159.5 | - |  |  |  |  |
| 41 |  |  |  | 122.2 | 9.42 (bs) |  |
| 42 | 133.9 | - |  |  |  |  |
| 43 | 105.9 | 5.79 (bs)  6.36 (bs) |  |  |  |  |
| 44 | 162.7 | - |  |  |  |  |
| 45 |  |  |  | 118.8 | 9.90 (bs) |  |
| 46 | 129.4 | - |  |  |  |  |
| 47 | 111.4 | 5.71 (bs)  5.72 (bs) |  |  |  |  |
| 48 | 158.3 | - |  |  |  |  |
| 49 | 139.1 | - |  |  |  |  |
| 50 | 140.0 | 8.54 (s) |  |  |  |  |
| 51 | 161.1 | - |  |  |  |  |
| 52 |  |  |  | 119.3 | 10.65 (bs) |  |
| 53 | 133.7 | - |  |  |  |  |
| 54 | 102.9 | 5.82 (bs)  6.55 (m) |  |  |  |  |
| 55 | 164.9 | - |  |  |  |  |
| 56 |  |  |  | 129.9 | 7.64 (bs)  8.16 (bs) |  |


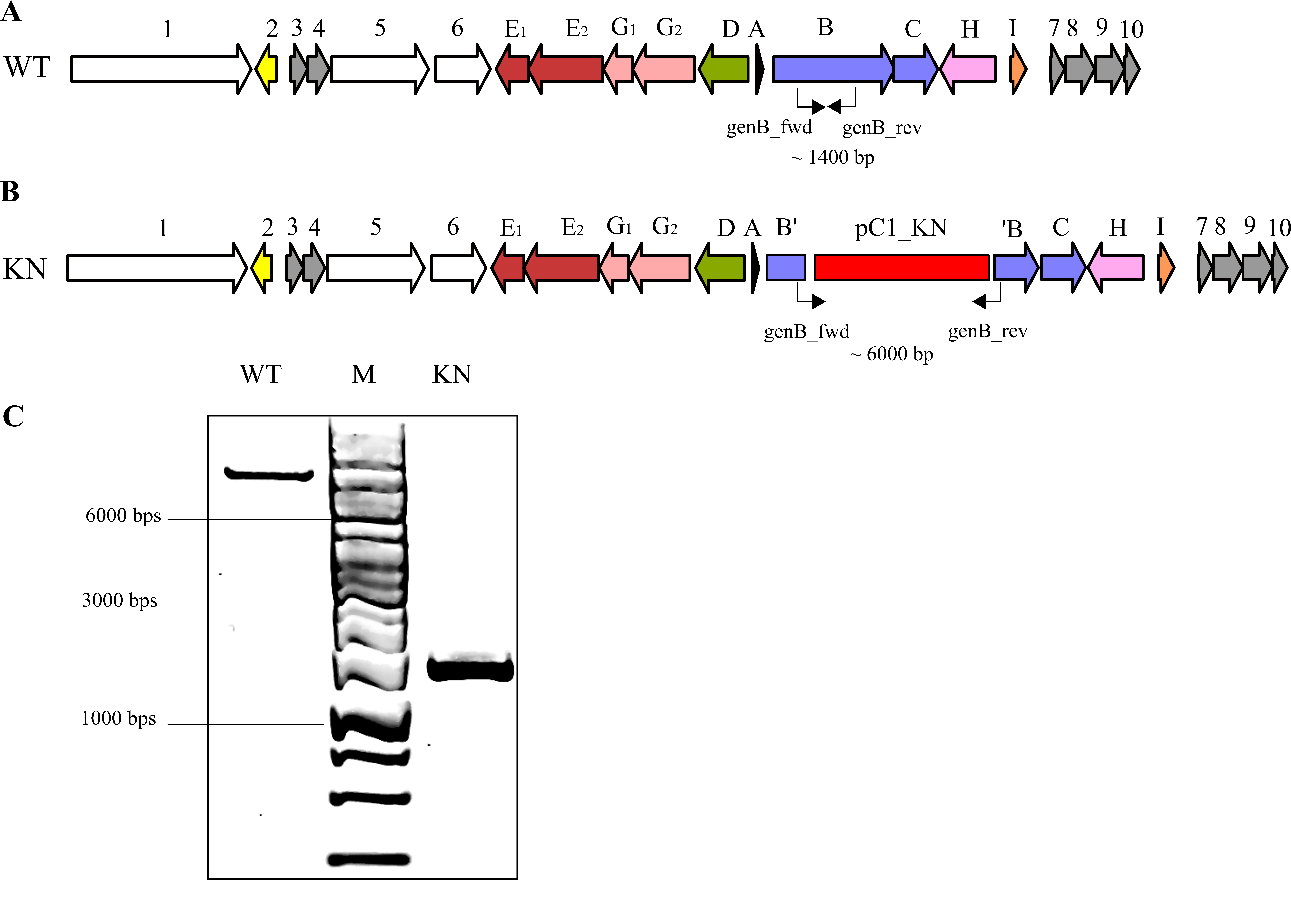


**Figure S1.** Schematic overview of the geninthiocin B BGC in YIM 130001 wild type (A) and knock-out mutant (B). Analytical PCR verification of pC1_KN insertion into the YIM 130001-genome (C) with genB_fwd/genB_rev primer set. WT: control, product from wild type YIM 130001 as template (ca 1400 bps); M: GeneRuler 1 kb DNA Ladder (ThermoFisher Scientific); KN: PCR product using genomic DNA from knock-out mutant YIM 130001/KN as template (ca 6000 bps).


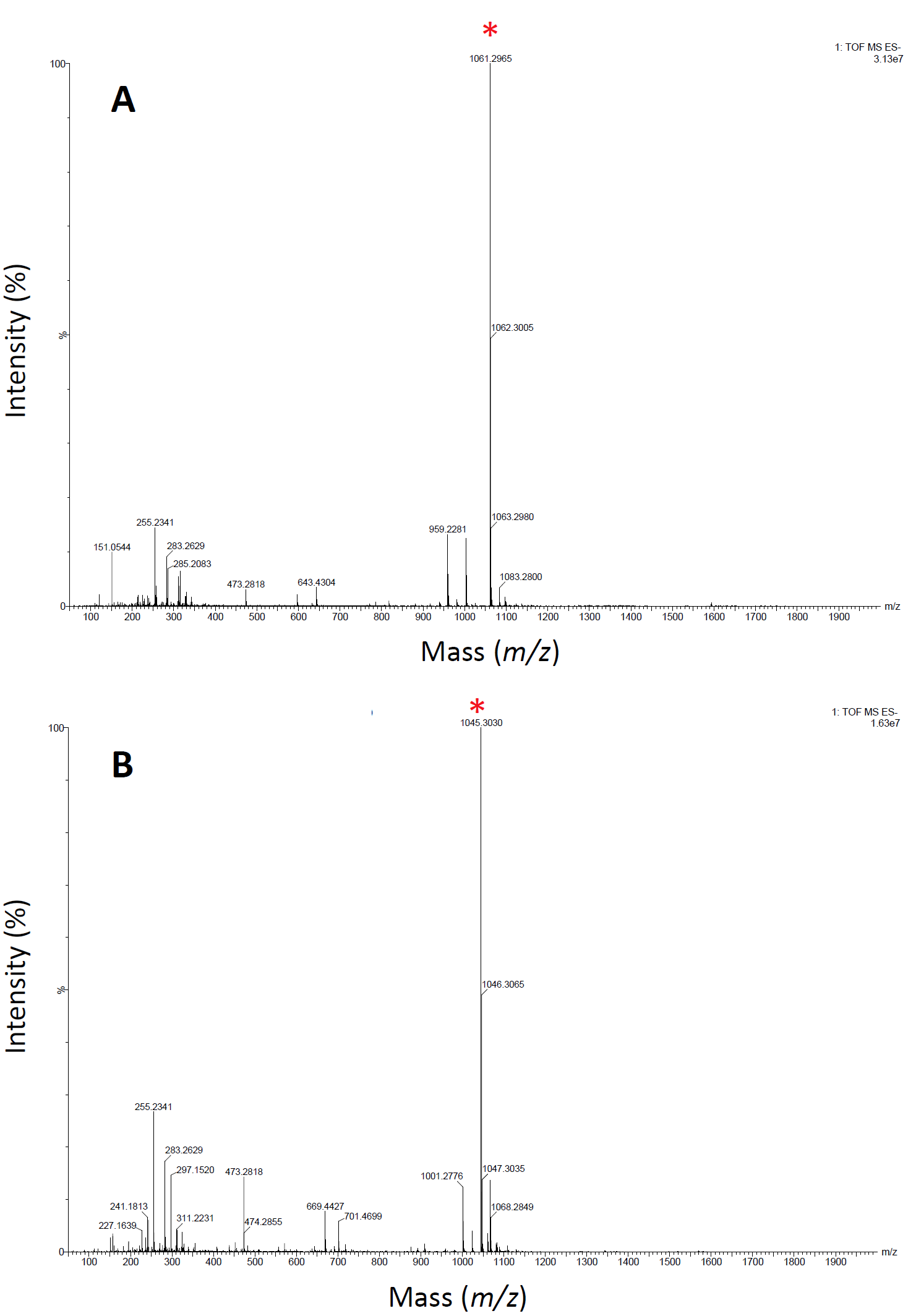


**Figure S2.** Direct flow injection analysis FIA-ESI-MS spectra obtained in negative ion mode from geninthiocin B (A) and compound 2 (B) after separation via preparative HPLC with respective masses (*).

**Figure S3**. Structure of the 35 macrocycle-membered geninthiocin B. For assigned ^1^H, ^13^C and protonated ^15^N shifts of peptide see Table S4.


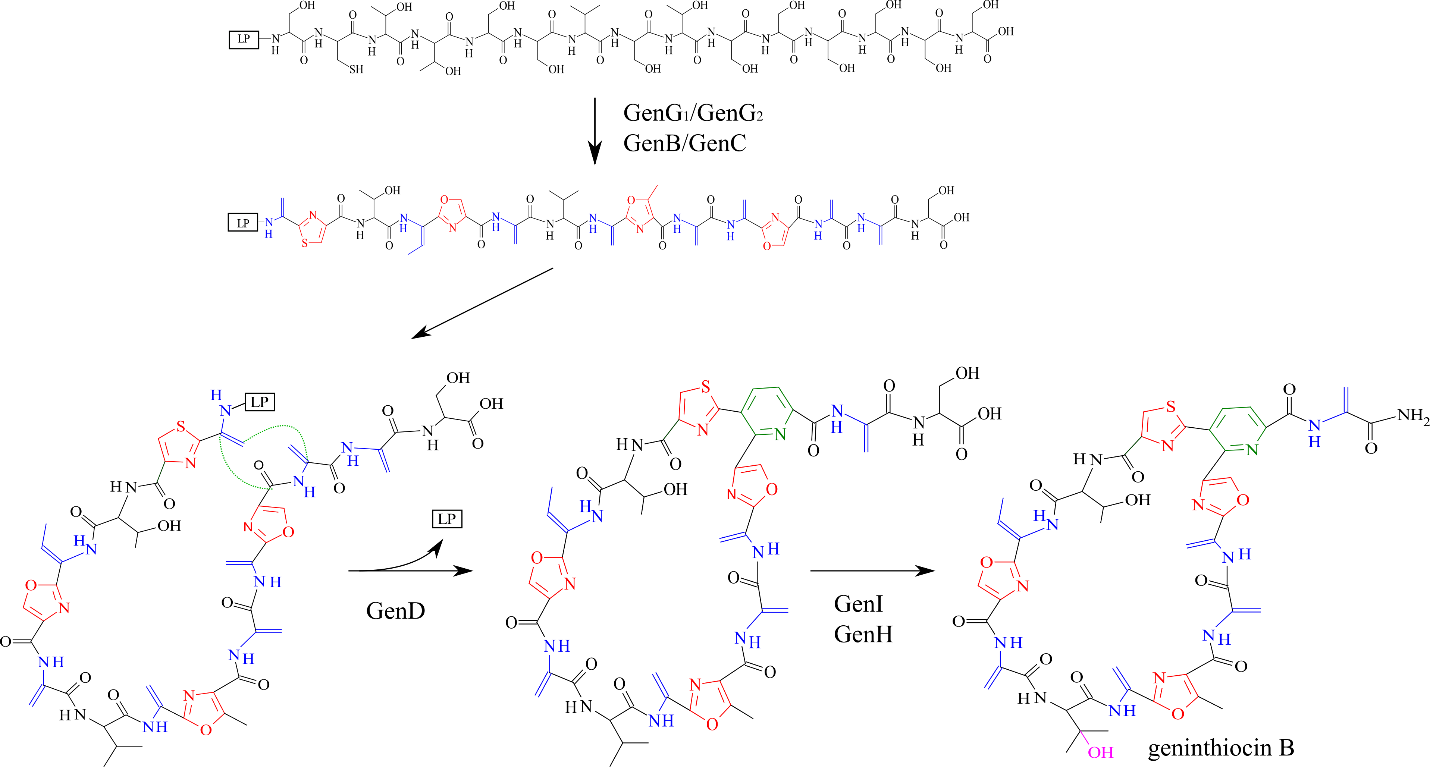


**Figure S4**. Proposed biosynthesis of geninthiocin B. Precursor pepide GenA contains a 31-aa leader peptide (LP) linked to a C-terminal 15-aa core peptide. In the first step of posttranslational modification proteins GenG1 and GenG2 will catalyse the processing of azole rings formation, whereas GenB and GenC, which showed high similarity to lanthipeptide-like dehydratases, will catalyse the formation of dehydroalanine (Dha) and dehydrobutyrine (Dhb). Two Dha groups from Ser_1_ and Ser_13_ will be utilized by GenD for assembly of the central six-membered nitrogenous heterocycle. The cleavage of Ser_15_ to afford the C-terminus amide and the hydroxylation of Val_7_ will be catalysed most probably by GenI and GenH, respectively.

**References**

1. Sekurova ON, Zhang J, Kristiansen KA, Zotchev SB. 2016. Activation of chloramphenicol biosynthesis in *Streptomyces venezuelae* ATCC 10712 by ethanol shock: insights from the promoter fusion studies. Microb Cell Fact. 15:85.
2. Zotchev S, Haugan K, Sekurova O, Sletta H, Ellingsen TE, Valla S. 2000. Identification of a gene cluster for antibacterial polyketide-derived antibiotic biosynthesis in the nystatin producer *Streptomyces noursei* ATCC 11455. Microbiology 146:611-619.
3. Flett F, Mersinias V, Smith CP. 1997. High efficiency intergeneric conjugal transfer of plasmid DNA from *Escherichia coli* to methyl DNA-restricting streptomycetes. FEMS Microbiol Lett 155:223-229.

1. * Shifts for protonated ^15^N have been determined from ^15^N HSQC experiment. Non-protonated ^15^N-atoms have not been assigned. [↑](#footnote-ref-1)
