# Supplementary material for: Genome Mining of Streptomyces sp. YIM 130001 Isolated From Lichen Affords New Thiopeptide Antibiotic
Source: Front Microbiol. 2018 Dec 19;9:3139. doi: 10.3389/fmicb.2018.03139 (PMC6306032; doi:10.3389/fmicb.2018.03139)
Supplement: Supplementary file 2 [file Data_Sheet_2.docx]

**Supplemental Material, NMR experiments**

**Genome Mining of *Streptomyces* sp. YIM 130001 Isolated from Lichen Affords new Thiopeptide Antibiotic**

Olha Schneider^1^, Nebojsa Simic^2^, Finn Lillelund Aachmann^1^, Christian Rückert^3^, Kåre Andre Kristiansen^1^, Jörn Kalinowski^3^, Yi Jiang^4^, Lisong Wang^5^, Cheng-lin Jiang^4^, Rahmi Lale^1^, Sergey B. Zotchev*^6^

**NMR of the peptide molecule**

The sample was prepared by dissolving 1.5 mg of compound in 160 µL DMSO-d6 (99.9% d) and transferred to a 3 mm NMR tube. All homo- and heteronuclear NMR spectra were recorded on a Bruker Ascend 800 MHz Avance III HD NMR spectrometer (Bruker BioSpin AG, Fälladen, Switzerland), equipped with 5 mm cryogenic CP-TCI probe. All NMR recording were performed at 25 °C. Shifts were determined relative to TMS, using the residual DMSO signals for spectra calibration. For chemical shift assignment of the compound, the following spectra were recorded: 1D proton, 1D ^13^C, 2D double quantum filtered correlation spectroscopy (DQF-COSY), 2D rotating-frame nuclear Overhauser effect correlation spectroscopy (ROESY) with 250 ms mixing time, 2D ^13^C heteronuclear single quantum coherence (HSQC) with multiplicity editing, 2D ^13^C HSQC-[^1^H,^1^H]TOCSY with 70 ms mixing time on protons, 2D heteronuclear multiple bond correlation (HMBC) with BIRD filter to suppress first order correlations and 2D ^15^N HSQC. The spectra were recorded, processed and analyzed using TopSpin 3.5 software (Bruker BioSpin) and provided in Figures 4-8.

The NMR structure elucidation showed clearly that the molecule contained a free primary amido-group (N-56, Fig.1) and 8 peptide bonds (Table 1 and Fig. 1). In addition, 5 terminal double bonds indicated 5 didehydroalanine residues. Threonine residue, didehydrobutyrine and hydroxy valine were also easily recognized. Identification of cyclic structures of thiazol, oxazol and pyridine were a bit more challenging, because of relatively large number of non-protonated carbons/heteroatoms and distant protons. Protons from the two OH groups could not be detected and assigned, probably because of their strong involvement in H-bonding. The shift assignments of ^1^H,^13^C and ^15^N (protonated) are shown in Table 1. The connectivity between the identified fragments was established primarily by HMBC experiment, and at less extent by DQF-COSY. The maps of HMBC and DQF-COSY correlations are shown in Fig. 2 and 3, respectively, in the supplement material.

Configurations at chiral C-atoms 11,12 and 28 were determined by combining data from 1D proton and ROESY experiments and analyses of the 3D simulated configuration of the molecule, obtained by MolView online application (molview.org).

**Configuration at C-11.** Based on the coupling constant for H-10 (doublet, 8.8 Hz), and the fact that H-10 has to be in the peptide plane, there are only two possible conformations for H-11, which would correspond to this magnitude of the coupling constant: *anti*, or *syn.* The ROESY correlation between methyl H-18 and methyl H-13 protons suggests an *anti*-conformation of H-11. The s*yn*-conformation would place the two methyl groups too far from each other for ROESY correlation to be observed. In addition, energy optimized 3D simulation of the molecule shows that the *syn*-conformation is less likely, due to considerable steric interactions. Therefore, the absolute configuration at C-11 has been determined as ***R*.**

**Configuration at C-12.** Since the proton signal at C-11 is a doublet of doublets, with coupling constants of 8.8 and 3.3 Hz, it is clear that the lower coupling constant corresponds to coupling with H-12. That fact implies that the relative conformation of H-11 and H-12 has to be *gauche*. Having in mind previously established conformation of methyl groups 18 and 13, the absolute configuration of C-12 has been determined as ***S*.**

**Configuration at C-28.** Proton H-27 is a doublet, with a coupling constant of 8.4 Hz. It is placed in the peptide bond plane. The magnitude of the coupling constant suggests *anti-* conformation of protons H-27 and H-28. Thus, the C-28 configuration has been determined as ***R***.

Table 1. Assigned ^1^H, ^13^C and protonated ^15^N shifts of peptide

| **No.** | **δ ^13^C** | **δ ^1^H** |  | **δ ^15^N^[[1]](#footnote-1)^*** | **δ ^1^H** |  |
| --- | --- | --- | --- | --- | --- | --- |
| 1 | 146.8 | - |  |  |  |  |
| 2 | 130.3 | - |  |  |  |  |
| 3 | 140.9 | 8.53 (d, *J*=8.1) |  |  |  |  |
| 4 | 121.5 | 8.25 (d, *J*=8.1) |  |  |  |  |
| 5 | 149.5 | - |  |  |  |  |
| 6 | 163.1 | - |  |  |  |  |
| 7 | 126.9 | 8.50 (s) |  |  |  |  |
| 8 | 149.4 | - |  |  |  |  |
| 9 | 159.9 | - |  |  |  |  |
| 10 |  |  |  | 107.9 | 8.03 (d, *J*=8.8) |  |
| 11 | 57.8 | 4.61 (dd, *J*=3.3, 8.8) |  |  |  |  |
| 12 | 67.3 | 4.29 (m) |  |  |  |  |
| 13 | 20.5 | 1.15 (d, *J*=6.3) |  |  |  |  |
| 14 | 168.8 | - |  |  |  |  |
| 15 |  |  |  | 117.0 | 9.66 (bs) |  |
| 16 | 123.1 | - |  |  |  |  |
| 17 | 129.6 | 6.56 (m) |  |  |  |  |
| 18 | 13.8 | 1.75 (d, *J*=7.1) |  |  |  |  |
| 19 | 159.4 | - |  |  |  |  |
| 20 | 142.8 | 8.71 (s) |  |  |  |  |
| 21 | 136.1 | - |  |  |  |  |
| 22 | 158.4 | - |  |  |  |  |
| 23 |  |  |  | 123.0 | 9.39 (bs) |  |
| 24 | 133.4 | - |  |  |  |  |
| 25 | 103.8 | 5.89 (bs)  6.46 (bs) |  |  |  |  |
| 26 | 163.7 | - |  |  |  |  |
| 27 |  |  |  | 108.9 | 8.30 (d, *J*=8.4) |  |
| 28 | 61.8 | 4.64 (d, *J*=8.4) |  |  |  |  |
| 29 | 74.0 | - |  |  |  |  |
| 30 | 26.2 | 1.21 (s) |  |  |  |  |
| 31 | 27.3 | 1.22 (s) |  |  |  |  |
| 32 | 169.4 | - |  |  |  |  |
| 33 |  |  |  | 128.1 | 9.70 (bs) |  |
| 34 | 128.6 | - |  |  |  |  |
| 35 | 105.7 | 5.65 (bs)  6.11 (bs) |  |  |  |  |
| 36 | 155.2 | - |  |  |  |  |
| 37 | 129.2 | - |  |  |  |  |
| 38 | 154.5 | - |  |  |  |  |
| 39 | 11.5 | 2.62 (s) |  |  |  |  |
| 40 | 159.5 | - |  |  |  |  |
| 41 |  |  |  | 122.2 | 9.42 (bs) |  |
| 42 | 133.9 | - |  |  |  |  |
| 43 | 105.9 | 5.79 (bs)  6.36 (bs) |  |  |  |  |
| 44 | 162.7 | - |  |  |  |  |
| 45 |  |  |  | 118.8 | 9.90 (bs) |  |
| 46 | 129.4 | - |  |  |  |  |
| 47 | 111.4 | 5.71 (bs)  5.72 (bs) |  |  |  |  |
| 48 | 158.3 | - |  |  |  |  |
| 49 | 139.1 | - |  |  |  |  |
| 50 | 140.0 | 8.54 (s) |  |  |  |  |
| 51 | 161.1 | - |  |  |  |  |
| 52 |  |  |  | 119.3 | 10.65 (bs) |  |
| 53 | 133.7 | - |  |  |  |  |
| 54 | 102.9 | 5.82 (bs)  6.55 (m) |  |  |  |  |
| 55 | 164.9 | - |  |  |  |  |
| 56 |  |  |  | 129.9 | 7.64 (bs)  8.16 (bs) |  |

**Acknowledgments**

This work was partly supported by the Research Council of Norway through the Norwegian NMR Platform, NNP (226244/F50).

Figure 1. The structure of the peptide

Figure 2. The map of HMBC correlations

Figure 3. The map of DQF-COSY correlations

Figure 4. 1D-^1^H NMR proton spectrum.

Figure 5. 1D ^13^C NMR spectrum.

Figure 6. ^1^H-^13^C HSQC NMR spectrum.

Figure 7. ^1^H-^15^N HSQC NMR spectrum.


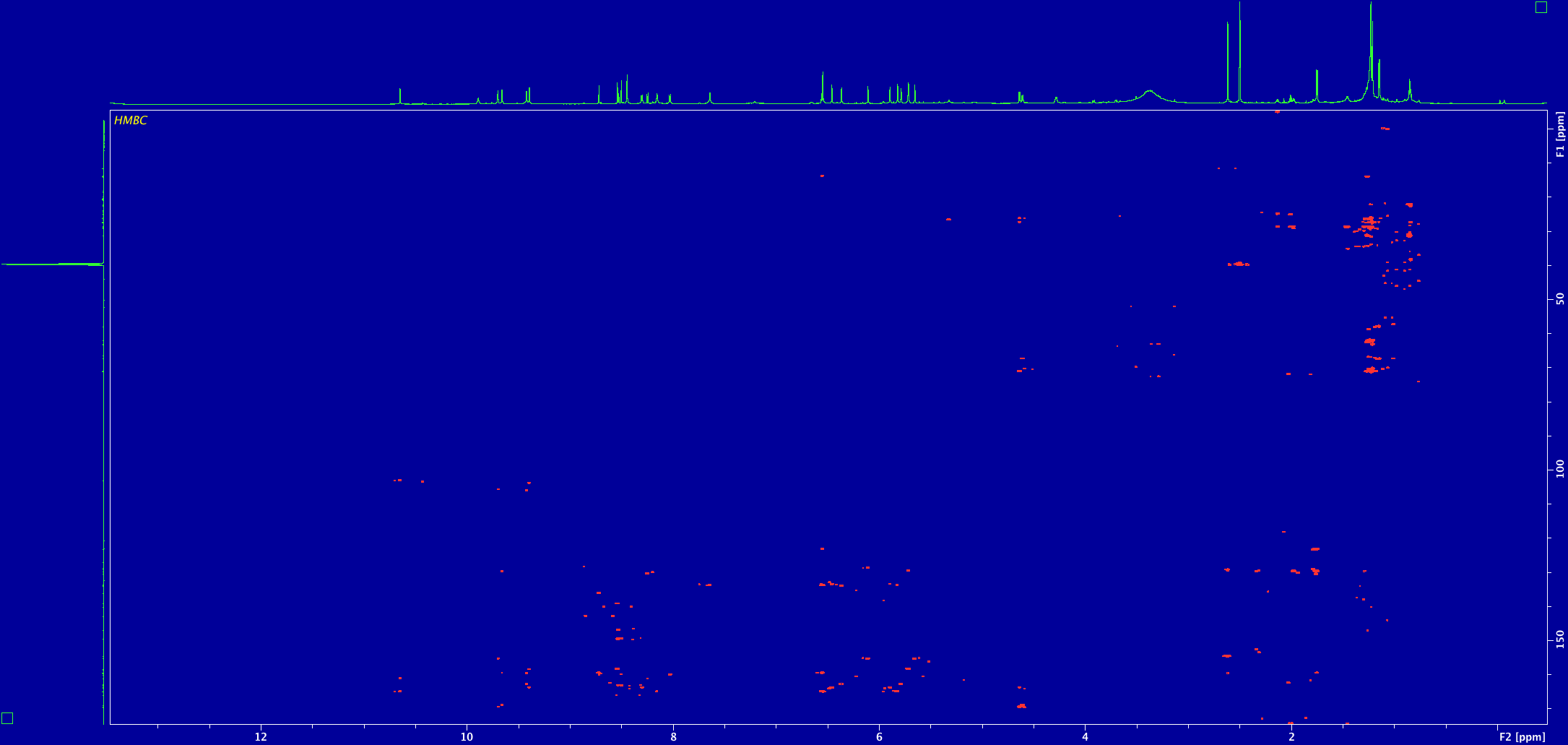


Figure 8. 2D heteronuclear multiple bond correlation (HMBC) with BIRD filter.

1. * Shifts for protonated ^15^N have been determined from ^15^N HSQC experiment. Non-protonated ^15^N-atoms have not been assigned. [↑](#footnote-ref-1)
